# To the Editors of the Medical and Physical Journal

**Published:** 1804-10-01

**Authors:** Frederic Thackeray

**Affiliations:** Cambridge


### *To the Editors of the Medical and Physical Journal.*

GENTLEMEN,

AS Mr. Goldson's pamphlet has excited some doubts in the breasts of many, as to the permanence of the vaccine influence, I have sent you the following experiment, which, indeed, has been made by hundreds before; but as nothing will tend more to silence the objection of its enemies, and confirm the expectations of the friends of the cow-pox, than a publication of facts, which prove its security, I should hope that from every quarter vacciolation will receive a support which its importance demands, and in which every friend of humanity is interested.

In the year 1801, a gentleman inoculated his own two boys and two of his servants with vacciolous matter, that was sent by Dr. Pearson; on the eighth, tenth, and twelfth days I saw their arms, and was well satisfied that vacciolation had taken place. Since then, they have frequently been exposed to variolous contagion, and the parent was convinced it could have no effect on his family, till Mr. Goldson's pamphlet raised his fears. To ease his mind, I inoculated with variolous matter his two boys, one of the servants, and two others who had never been inoculated in any way. The arms of the boys and the servant inflamed from the third to the seventh day, when it gradually subsided, and on the ninth no mark was visible; in the two others, the small-pox pursued its usual course; the eruptions indeed were not numerous, though the eruptive fever was troublesome for two days. There is no occasion for comment, as the fact speaks for itself.

I am, &c.

FREDERIC THACKERAY.

*Cambridge, September* 4, 1804.

P. S. After numberless inquiries in this county, I have never but once met the cow-pox in the cow itself; and it was deemed such a curiosity in the parish, that I was requested to see it; it belonged to the blacksmith. He could assign no reason; and told me, that he had seen no horse with the scratchy heel for some time. As the herd passed by, I observed, that the bull had his foot tied up, in consequence of a sore in the hoof. They who believe that the greasy heel of the horse is instrumental in producing vacciola, ciola, may think that a similar matter may be secreted in the ulcerated hoof of the bull; and that, in the above instance, the dug of the cow had rested where the diseased foot of the bull had trodden.

---

